# Interfacial energy constraints are sufficient to align cells over large distances

**DOI:** 10.1016/j.bpj.2025.02.011

**Published:** 2025-03-12

**Authors:** Sham Tlili, Murat Shagirov, Shaobo Zhang, Timothy E. Saunders

**Affiliations:** 1 Mechanobiology Institute, National University of Singapore, Singapore, Singapore; 2 Aix-Marseille University, CNRS, UMR 7288, IBDM, Turing Center for Living Systems, Marseille, France; 3 Department of Biological Sciences, National University of Singapore, Singapore, Singapore; 4 Institute of Molecular and Cell Biology, A^∗^Star, Singapore, Singapore; 5 Warwick Medical School, University of Warwick, Coventry, UK

## Abstract

During development and wound healing, cells need to form long-range ordered structures to ensure precise formation of organs and repair damage. This requires cells to locate specific partner cells to which to adhere. How such cell matching reliably happens is an open problem, particularly in the presence of biological variability. Here, we use an equilibrium energy model to simulate how cell matching can occur with subcellular precision. A single parameter—encapsulating the competition between selective cell adhesion and cell compressibility—can reproduce experimental observations of cell alignment in the *Drosophila* embryonic heart. This demonstrates that adhesive differences between cells (in the case of the heart, mediated by filopodia interactions) are sufficient to drive cell matching without requiring cell rearrangements. The biophysical model can explain observed matching defects in mutant conditions and when there is significant biological variability. Using a dynamic vertex model, we demonstrate the existence of an optimal range of effective cell rigidities for efficient matching. Overall, this work shows that equilibrium energy considerations are consistent with observed cell matching in cardioblasts and has potential application to other systems, such as neuron connections and wound repair.

## Significance

Cells often need to identify specific neighboring cells, such as during wound repair and forming neural connections. Here, we develop a biophysical model of such cell-cell interactions within the context of the developing heart. We demonstrate that precise cell matching can occur by minimizing the energy costs of interfacial interactions. This model can explain a breadth of experimental observations despite it being a steady-state approximation of a dynamic system. This opens the possibility that such approaches may be applicable to other systems, providing a powerful yet simple framework for understanding cell matching.

## Introduction

During development, cells interact collectively to form tissues and organs through a series of morphological transformations driven by cell proliferation, rearrangements, migration, and death (1,2,3,4,5). When these processes fail, the final shape of the tissue can be defective, resulting in diseases, including cardiomyopathies (6,7) and neurological defects (8). During organogenesis, cells often need to identify specific cells to which to adhere (9). A classic example is formation of human facial structures (8,10); cells initially undergo long-range migration from distinct regions of the neural plate before forming precise connections to create structures such as the lip. Errors in this process lead to birth defects such as cleft lip and facial cleft (10). During neurogenesis, neurons also need to form precise linkages to their synaptic partners (11,12,13) with severe consequences if these processes fail. A range of molecules have been identified that are involved in cell matching, predominantly from neuronal systems (14,15,16,17). These molecules include cytoskeletal, adhesion, and force-transducing proteins (9). However, the underlying mechanisms by which the information from these different components is integrated by the developing tissue to form precise connections remain unknown.

In most tissues, there are multiple cell types with stereotypic spatial positions. For example, formation of the eye requires precise cell fate determination and positioning (18,19), even within a growing domain (20). In the developing heart, cardioblasts take on different fates depending on expression of (highly conserved) transcription factors (21). Periodic patterns of cells can also be generated from lateral inhibition (22,23). Such periodic patterns need to be maintained across large distances, even as tissues undergo large-scale morphological changes. Experimental and theoretical work has begun to reveal how neighboring tissues can form distinct boundaries (24,25). An important concept in generating and maintaining tissue structural organization is the differential adhesion hypothesis (26,27). By cells mechanically interacting differently with each other, depending on specific fate, cells can form ordered structures and patterns.

From flies to humans, early heart development involves the migration of two contralateral rows of cells to the embryonic midline (9). There, they form the first linear heart tube, with a lumen, capable of pumping fluid (28). In *Drosophila*, the lumen initially forms between cell doublets, so the contralateral neighbors need to be aligned. For the heart to pump fluid requires the cardioblast to generate a continuous lumen. In previous work, we demonstrated that, when cell alignment failed, the heart was unable to pump correctly (29). The adhesion molecules Fasciclin3 (Fas3) and Teneurin-m (Ten-m) are critical in ensuring this robust alignment (29). In humans, errors in the process of heart alignment can lead to diseases such as cardia bifida (30).

Theoretical modeling of tissue formation has helped increase our understanding of how cells pack (31), form compartment boundaries (32), generate complex tissue shapes (33,34,35,36,37), and ensure regulated growth (38). Lateral inhibition can create a wide variety of patterns depending on the feedback mechanisms (39). Recently, vertex models have been used to understand cell structure in epithelia (40,41), including in three dimensions (42). Although biological systems are inherently dynamic, equilibrium statistical mechanics can be a powerful tool for understanding suitable biological processes. An example of such a case is cell packing in the eye, where analogies with soap bubbles provide an effective tool for understanding formation of the *Drosophila* retina (43).

**Figure 1:**
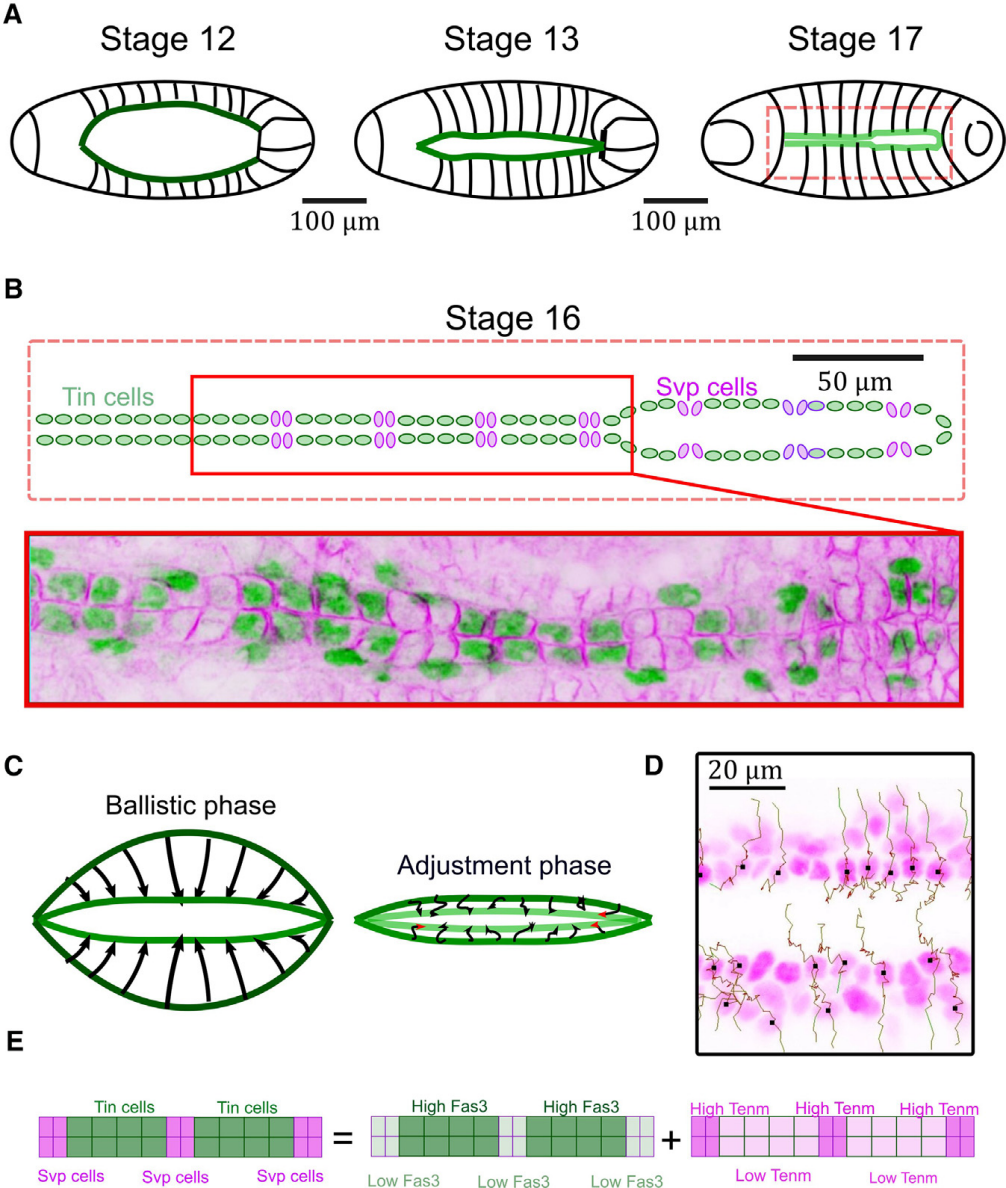
Cell matching in *Drosophila* embryonic heart. (*A*) Developmental stages of heart formation. Cardioblasts (*green regions*) merge to form a tubular heart (stage 17). (*B*) Cardioblasts have distinct expression patterns. Heart structure at stage 16: the heart is made of a periodic alternation of Tin cells and Svp cells. Staining of cardioblasts (Spectrin in magenta and Tinman in green). (*C*) Heart formation is initially driven by a global tissue movement (a ballistic phase in which cardiac cells are passively driven by the dorsal closure process). Once the two contralateral rows of cardioblasts are almost in contact, active processes align cells in a final adjustment phase. (*D*) Example of cell trajectories during the ballistic (straight trajectories) and adjustment phase (diffusive trajectories). (*E*) Cardioblasts express the adhesion molecules Fas3 and Ten-m in an alternating pattern.

Here, we develop a model of cell matching, where differential adhesion energy constraints between cells drives the process of matching. We apply this to the developing *Drosophila* embryonic heart, where the process of cardioblast cell matching has been quantified (29,44,45). The *Drosophila* embryonic heart is composed of two lines of cardioblasts that migrate together over a period of a few hours (Fig. 1 *A*), and they express either Tinman (Tin, the *Drosophila* homolog of mammalian Nkx2.5) or Seven-up (Svp), Fig. 1 *B*, in a repeating 4-2 pattern (46). Cardioblasts first approach each other through migration toward the embryo midline, driven by surrounding tissues (47) and myosin contractility (48), which we refer to as a ballistic phase, Fig. 1 *C*. As cardioblasts approach each other, the cells adjust position, via filopodia interactions between cardioblasts, to align accurately with their contralateral partners (Fig. 1 *D*). Specific adhesion molecules are expressed within the different cardioblast types: Fas3 in Tin-positive cardioblasts and Ten-m in Svp-positive cardioblasts (Fig. 1 *E*). Cell matching in the heart depends on the differential spatial expression of these adhesion molecules (29). The mechanical force between opposing cells is generated by a periodic wave of Myosin II in the cardioblasts, which acts to “proof read” the mechanical connections (44,45). Motivated by these observations, we construct a biophysical model of the cell-cell interactions to test whether such differential adhesion is sufficient to drive cell matching. We then use this model to test how both structural and genetic perturbations alter cell matching. This model provides a biophysical framework in which to understand how cells within confluent tissues find specific partners during development.

## Materials and methods

### Methods

Experimental data shown are taken from (29,44). A detailed methodology for the quantification of heart cell matching is available (45).

### Modeling

#### One-dimensional interface and mismatch ratio definitions

**Figure 2:**
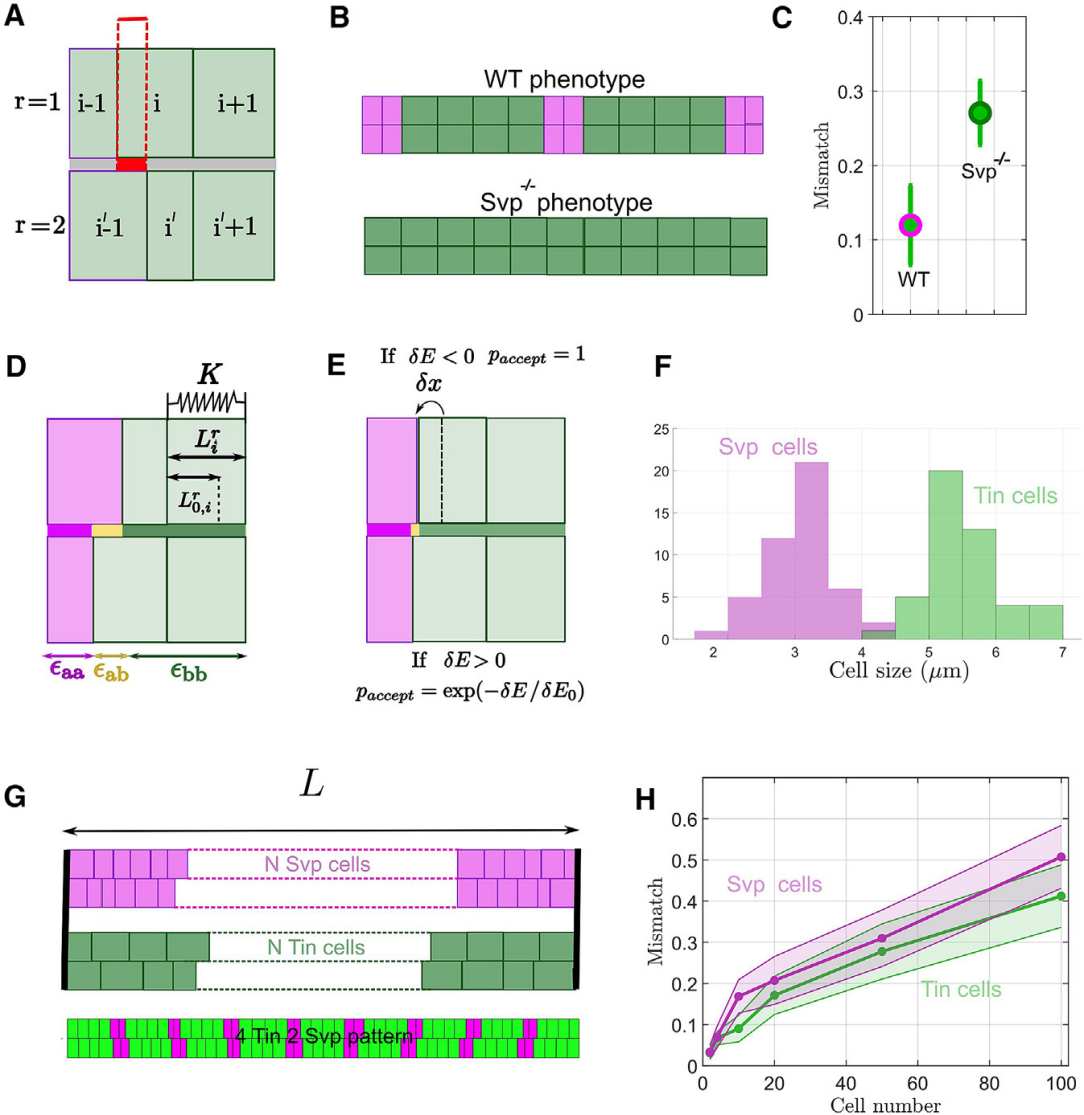
Simulating heart matching. (*A*) Definition of cell mismatch. r = 1,2 denotes two contralateral rows of cardioblasts. The dashed red box denotes a “mismatched” region in which two cells which are not supposed to be in contact are partially facing. (*B*) The wild-type heart (*top*) has a stereotypic pattern of cardioblast fates (denoted by magenta (Svp-positive) and green (Tin-positive) cells), which express specific adhesion molecules. Such a pattern is lost in *svp*^*−/−*^ mutants (bottom), where there is a single cardioblast type, Tin cells. (*C*) It has been experimentally shown by some of us that heart cell mismatch (as defined in *A*) is affected by the loss of adhesion pattern in *svp*^*−/−*^ mutants (29). (*D*) Considering two cell types (denoted by *a* (*magenta*) and *b* (*green*)), there are three interface energies: *ε*_aa_ between two cells of type a, *ε*_bb_ between two cells of type b, and *ε*_ab_ between cells of different type (denoted by yellow region). Cells can deform elastically (or compress), *L*_*i*_ from their rest length, *L*_*0*_ with a compressibility K, and cell-cell contact is characterized by an adhesion depending on the contact type. (*E*) To find how cell differential adhesion competes with the elastic deformations generated by local cell compression, a Metropolis algorithm is used to find the final cell alignment after equilibration. At each step, a cell/cell interface is randomly chosen and is displaced by *δ*x, which follows a Gaussian distribution (see materials and methods). The probability to accept the displacement follows the Metropolis algorithm. (*F*) Experimental distribution of leading-edge size in different cardioblast types (29). (*G*) Simulations with only one cell type (all Svp or all Tin) and with the alternating pattern of Tin/Svp cells (*bottom*). (*H*) Predicted mismatch in hearts with one cell type for different cell number. The mismatch increases with the linear size of the system (the number of cells in a heart row) as each cell width varies as a random variable.

The model system consists of two rows of equal number of cells interacting on a one-dimensional interface defined as vertices x, Fig. 2 *A*. Each cell i on a row r is represented by a pair of vertices $\left(x_{i}^{\left(r\right)},x_{i+1}^{\left(r\right)}\right)$ on this interface with $x_{i}^{\left(r\right)}\leq x_{i+1}^{\left(r\right)}$ *(*i=1,2,…,N+1, and r∈{1,2} for N cells occupying each row), with cell length

(1) $$L_{i}^{\left(r\right)}=x_{i+1}^{\left(r\right)}-x_{i}^{\left(r\right)}$$

The total length of a model system is kept constant by fixing first $x_{1}^{\left(1\right)}=x_{1}^{\left(2\right)}$ and last $x_{N+1}^{\left(1\right)}=x_{N+1}^{\left(2\right)}$ vertices throughout the simulation. The overlap interval length between cells i and j located on two different rows is calculated as

(2) $$L\left(i\cap j\right)=\left\{ \begin{array}{c} 0,\text{if}\;\max\left(x_{i}^{\left(1\right)},x_{j}^{\left(2\right)}\right)>\min\left(x_{i+1}^{\left(1\right)},x_{j+1}^{\left(2\right)}\right)\\ \min\left(x_{i+1}^{\left(1\right)},x_{j+1}^{\left(2\right)}\right)-\max\left(x_{i}^{\left(1\right)},x_{j}^{\left(2\right)}\right),otherwise \end{array}\right.$$

The mismatch ratio $m_{i}^{\left(r\right)}$ of a cell i on a row r is defined as a proportion of length of overlap with cells other than its sister cell $i^{\prime }$, and the total cell length of i:

(3) $$m_{i}^{\left(r\right)}=\frac{L_{i}^{\left(r\right)}-L\left(i\cap i^{\prime }\right)}{L_{i}^{\left(r\right)}};\;m_{i}^{\left(r\right)}\in \left[0,1\right]$$

where the sister cell is defined as a cell $i^{\prime }$ with same index as i but located on a different row (i.e., $i^{\prime }=i$), an average mismatch ratio of two sister cells is then calculated as $\left(m_{i}^{\left(1\right)}+m_{i^{\prime }}^{\left(2\right)}\right)/2$.

#### Calculating net change in energy

All vertices $x_{i}^{\left(r\right)}$ are assumed to be coupled with harmonic springs, with each cell having an elastic energy $E_{\text{el},i}^{\left(r\right)}=K\cdot {\left(L_{i}^{\left(r\right)}-L_{0,i}^{\left(r\right)}\right)}^{2}$, where $L_{0,i}^{\left(r\right)}$ is the equilibrium length for a given cell (kept constant throughout the simulation) and K is a spring constant. Then, the total elastic energy for the whole system is just a sum of elastic energies over all cells:

(4) $$E_{\text{el},\text{total}}=\sum_{r=1}^{2}\sum_{i=1}^{N}K\cdot {\left(L_{i}^{\left(r\right)}-L_{0,i}^{\left(r\right)}\right)}^{2}$$

In the model, two rows of cells interact along the one-dimensional interface by adhesion of cells along segments of the interface shared by the same cell type cells or by cohesion of cells of two different cell types along interface segments occupied by cells of two different types. We implemented the interface by defining interface vertices $x_{j}^{\left(\mathrm{int}\right)}$ with $x_{j}^{\left(\mathrm{int}\right)}\leq x_{j+1}^{\left(\mathrm{int}\right)}$, which could be obtained by concatenating both rows of $x_{i}^{\left(r\right)}$ vertices into single vector and then sorting them. Similar to cell vertices *(*$x_{i}^{\left(r\right)}$*)*, pair of interface vertices $\left(x_{j}^{\left(\mathrm{int}\right)},x_{j+1}^{\left(\mathrm{int}\right)}\right)$ represents an interface segment j. The total adhesion energy for the whole system is then the sum of adhesion energy contributions of all interface segments:

(5) $$E_{adh,total}=\sum_{j=1}^{2N+1}\epsilon_{j}\cdot \left(x_{j+1}^{\left(\mathrm{int}\right)}-x_{j}^{\left(\mathrm{int}\right)}\right)$$

where $\epsilon_{j}$ is the cell-type-specific adhesion energy per unit length of a segment j:

(6) $$\epsilon_{j}=\left\{ \begin{array}{c} \epsilon_{aa},j\;\text{is}\;\text{interface}\;\text{between}\;\text{type}\;a\;\text{and}\;a\\ \epsilon_{bb},j\;\text{is}\;\text{interface}\;\text{between}\;\text{type}\;b\;\text{and}\;b\\ \epsilon_{ab},j\;\text{is}\;\text{interface}\;\text{between}\;\text{type}\;a\;\text{and}\;b \end{array}\right.$$

Since vertices of $x_{j}^{\left(\mathrm{int}\right)}$ are from both rows of cells, changing position of a single cell vertex $x_{i}^{\left(r\right)}$ could result in cases with $x_{j}^{\left(\mathrm{int}\right)}\geq x_{j+1}^{\left(\mathrm{int}\right)}$, thus $x_{j}^{\left(\mathrm{int}\right)}$ needs to be sorted before calculating total adhesion energy during simulation.

The total energy of the system as a function of location of cell vertices $x_{i}^{\left(r\right)}$ is calculated as the sum of elastic and adhesive energies:

(7) $$E\left(x\right)=E_{\text{el},\text{total}}+E_{adh,total}$$

and the net change in total energy due to change in the configuration of x→x+Δx is calculated as a difference in total energy between the two configurations

(8) $$\Delta E_{x\to x+\Delta x}=E\left(x+\Delta x\right)-E\left(x\right)$$

#### Sampling initial configuration and equilibrium lengths of cells

Randomness in cell shape geometry was modeled as a random initial configuration of cells, which was set to be equal to the equilibrium lengths of the cells throughout the simulation. The random initial configurations of cells, and thus the equilibrium lengths ($L_{0,i}^{\left(r\right)}$) for each cell, were sampled from a normal distribution with cell-type-specific mean and standard deviation using a MATLAB function *normrnd*. To construct a random configuration, we implemented random sequential deposition (49) of two rows onto each other with a constraint that enforces equal length of two rows within $\varepsilon =\mu \cdot 10^{-3}$, where μ is the smallest of the two type-specific average lengths of the cells in the given configuration.

For the row of cells with $m_{a}$ type a cells and $N-m_{a}$ type b cells, $m_{a}$ equilibrium lengths ($L_{0,i}^{\left(1\right)}$) for the top row are sampled from a normal distribution with mean $L_{0,a}^{mean}$ and standard deviation $L_{0,a}^{std}$, and $N-m_{a}$ equilibrium lengths ($L_{0,i}^{\left(1\right)}$) are sampled from normal distribution with mean $L_{0,b}^{mean}$ and standard deviation $L_{0,b}^{std}$.

$$L_{0,i}^{\left(1\right)}∼\left\{ \begin{array}{c} N\left({\mu =L}_{0,a}^{mean},\sigma =L_{0,a}^{std}\right)\;\text{for}\;\text{type}\;a\;\\ N\left({\mu =L}_{0,b}^{mean},\sigma =L_{0,b}^{std}\right)\;\text{for}\;\text{type}\;b \end{array}\right.$$

where the mean and standard deviation for different cell types are experimentally determined parameters. Next, the bottom row equilibrium lengths ($L_{0,i}^{\left(2\right)}$) were sampled in the same manner until the constraint $\sum_{i}L_{0,i}^{\left(1\right)}-\varepsilon <\sum_{i}L_{0,i}^{\left(2\right)}<\sum_{i}L_{0,i}^{\left(1\right)}+\varepsilon$ was satisfied. Afterward, $L_{0,i}^{\left(r\right)}$ are converted into cell vertices $x_{i}^{\left(r\right)}$ in the same sequence as the $L_{0,i}^{\left(r\right)}$ was produced by *normrnd* function*.*

#### Implementation of Markov-chain cell configuration sampling using Metropolis algorithm

To simulate evolution of the cell boundaries by moving cell vertices x and sample cell configurations, we used Monte Carlo Markov-chain (MCMC) sampling by implementing the Metropolis algorithm in MATLAB (50). At each MCMC sampling step, a vertex $x_{i}^{\left(r\right)}$ is selected randomly and a random move is proposed $x_{i}^{\left(r\right)}\to x_{i}^{\left(r\right)}+\Delta x_{i}^{\left(r\right)}$; $\Delta x_{i}^{\left(r\right)}$ is a Gaussian random variable with mean 0 and standard deviation δX, and the value of δX determines magnitude of the random movement (interpreted as random fluctuations in the model). In our implementation, $\Delta x_{i}^{\left(r\right)}$ was sampled using the *normrnd(0,* δX*)* function in MATLAB. Afterward, Metropolis probability of accepting the proposed move, $p\left(x_{i}^{\left(r\right)}\to x_{i}^{\left(r\right)}+\Delta x_{i}^{\left(r\right)}\right)$ is calculated using

(9) $$p\left(x_{i}^{\left(r\right)}\to x_{i}^{\left(r\right)}+\Delta x_{i}^{\left(r\right)}\right)=\min\left(1,\exp\left(-\frac{\Delta E_{x_{i}^{\left(r\right)}\to x_{i}^{\left(r\right)}+\Delta x_{i}^{\left(r\right)}}}{E_{0}}\right)\right)$$

where $\Delta E_{x_{i}^{\left(r\right)}\to x_{i}^{\left(r\right)}+\Delta x_{i}^{\left(r\right)}}$ is a net change in total energy of the system due to movement of vertex $x_{i}^{\left(r\right)}$ by amount $\Delta x_{i}^{\left(r\right)}$, and $E_{0}$ is the effective temperature (“thermal energy”) of the cells. Then, $p\left(x_{i}^{\left(r\right)}\to x_{i}^{\left(r\right)}+\Delta x_{i}^{\left(r\right)}\right)$ is compared to a uniformly distributed random variable $\delta_{p}$ in the interval $\delta_{p}\in \left(0,1\right)$ produced by *rand* function in MATLAB. If the statement $\delta_{p}<p\left(x_{i}^{\left(r\right)}\to x_{i}^{\left(r\right)}+\Delta x_{i}^{\left(r\right)}\right)$ is true, then the move is accepted, and otherwise the move is rejected.

Overall, the probability of movement $x_{i}^{\left(r\right)}\to x_{i}^{\left(r\right)}+\Delta x_{i}^{\left(r\right)}$ of a vertex at position $x_{i}^{\left(r\right)}$ is

$$P\left(x_{i}^{\left(r\right)}\to x_{i}^{\left(r\right)}+\Delta x_{i}^{\left(r\right)}\right)=\frac{1}{2\cdot \left(N-1\right)}\cdot p\left(x_{i}^{\left(r\right)}\to x_{i}^{\left(r\right)}+\Delta x_{i}^{\left(r\right)}\right)$$

where the first term on the right-hand--side refers to the probability of selecting vertex $x_{i}^{\left(r\right)}$ from two rows, and N−1 vertices in each row (two end vertices on each row are assumed to be fixed throughout the simulation, and have P=0). To avoid flipping of cells, and overlapping of cells on a single row, we forbid such movements by setting $p\left(x_{i}^{\left(r\right)}\to x_{i}^{\left(r\right)}+\Delta x_{i}^{\left(r\right)}\right)=0$ for movements with $\Delta x_{i}^{\left(r\right)}>0$ that exceed cell length of cell i (i.e., $\Delta x_{i}^{\left(r\right)}>0\;\text{AND}\;\Delta x_{i}^{\left(r\right)}>L_{i}^{\left(r\right)}$), and for movements with $\Delta x_{i}^{\left(r\right)}<0$ that exceed cell length of cell i−1 (i.e., $\Delta x_{i}^{\left(r\right)}<0\;\text{AND}\;|\Delta x_{i}^{\left(r\right)}\left|\right\rangle L_{i-1}^{\left(r\right)}$). In practice, we used δX=0.05 and $\frac{E_{0}}{K}=0.1$. See Fig. S1 to see the impact of the effective temperature on the final mismatch.

#### Implementation of vertex model

Cell initial geometries are constructed using the same functions as with the equilibrium energy model above. Each vertex is described by its position $x_{i}^{\left(r\right)}\left(t\right)$ and follows the equation: $\xi \frac{\partial x_{i}^{\left(r\right)}\left(t\right)}{\partial t}=(T_{adh}^{i+1}-T_{adh}^{i}+K*\log\left(L_{i+1}^{\left(r\right)}\left(t\right)/L_{0,i+1}^{\left(r\right)}\right)-K*\log\left(L_{i}^{\left(r\right)}\left(t\right)/L_{0,i}^{\left(r\right)}\right)$ with $T_{adh}=T_{SS}$ for an S-S interface, $T_{adh}=T_{TT}$ for a T-T interface, and $T_{adh}=T_{ST}$ for an S-T interface. Here, we use the elastic force form K ^∗^log($L_{i}^{\left(r\right)}\left(t\right)/L_{0,i}^{\left(r\right)}$) instead of K ^∗^ ($L_{i}^{\left(r\right)}\left(t\right)-L_{0,i}^{\left(r\right)}$) to avoid numerical artifacts at big cell deformation. These two definitions are the same for small cell deformations. Time is discretized in time steps dt = 0.01 min and the friction parameter ξ order of magnitude is tuned to observe the dynamics of the system evolution on the minute/hour timescale. Our previous equilibrium model predicts that matching will occur when $K∽T_{adh}$ and on the hour timescale when $T_{adh}/\xi$ ∽1μm/min. In Fig. 6, $T_{adh}$ and K are expressed in pN and ξ in pN ^∗^min/μm. The vertices positions are calculated at each simulation step and allow us to calculate the mismatch evolution in time.

#### Interpretation of experimental measurements within the model space

In Fig. 4C, we map experimental observations of mismatch onto our model predictions. The two levels, dark or light, represent the wild-type expression levels of each adhesion molecule in the two cell types. Specifically, the S-type exhibits high Ten-m and low Fas3 expression, whereas the T-type displays high Fas3 and low Ten-m expression. In *fas3*^*−/−*^ and *ten-m*^*−/−*^ mutants, the levels of Fas3 and Ten-m are assumed to be strictly null in all cells. We validate that the sum of *γ* values obtained for these two conditions is equal to the *γ* value observed in the wild-type case. This result is consistent with a decomposition using the six parameters $E_{SS}^{T}$, $E_{TT}^{F},E_{ST}^{F},E_{ST}^{T},E_{TT}^{T},E_{SS}^{F}$. In the *svp*^*−/−*^ mutant and the double mutant, all cells exhibit the same Fas3 and Ten-m levels, leading to *γ* = 0. In the Svp-Gal4, Fas3-UAS condition (green diamond), the Fas3 level is assumed to be identical in both S and T cell types, although this is an approximation as we see quite a large amount of cell-to-cell variability (which is often the case with such Gal4-UAS knockdowns). In the Svp-Gal4 > Fas3-RNAi-UAS condition, the Fas3 level in Svp-type cells is reduced from its low level in the wild-type condition to a null level.

## Results

### Mapping of adhesive interactions onto an equilibrium energy state

To simulate cell matching, we utilized our energy-based model (materials and methods), which accounts for the spatial constraints between cells and the adhesion competition between different cell types. In the *Drosophila* heart, differential adhesion between cells is mediated by filopodia contacts, which have a spread of contact times, $\tau_{bind}$ (29,44), from a few seconds to over 5 min.

At a biochemical level, the time for reactions, τ, are typically related to the Arrhenius law, $\tau ∼{\tau_{0}e}^{\Delta E/k_{B}T}$, where ΔE is the activation energy. Timescales in biological systems at macroscopic levels often follow the Arrhenius law, such as in the developmental time of *Drosophila* embryogenesis (51,52). Here, we apply a *mesoscopic* approximation, where we assume that the distribution of binding times of filopodia $\tau_{bind}$ can be related to the effective adhesion activation energy ${\Delta E}_{adh}$. Underlying this model is the assumption that mechanical interactions between cells are mediated by filopodia and this is defining the relevant timescales. The essential ingredient is that such interactions are faster than the cell alignment time, therefore allowing an equilibrium assumption. The nature of the specific cell-cell mechanical interactions is not essential for the below results.

In the model, the overlap between facing cells results in an adhesion energy ΔE=−ϵ.x, where *x* denotes the length of the cell contact interface and ϵ is the adhesion energy per unit length between the two cells. In previous work (29), we showed that the expression of specific adhesion molecules is patterned along the heart (Fig. 2 *B*); disruption to this pattern leads to matching defects (Fig. 2 *C*). We can encode these adhesion differences as effective energy in our model (Fig. 2 *D*). Cellular compressibility for each cell is encoded by an effective elastic energy $E_{el}=K{\left(L_{cell}-L_{0}\right)}^{2}$, corresponding to the cost of deforming the cells away from their preferred cross-sectional width; K is the effective cell compressibility along its leading edge, $L_{cell}$ the cell length, and $L_{0}$ the cell rest length (Fig. 2 *D*). We only focus on the apical leading edge of the cells as this is where the process of cell alignment is occurring. Combining the energy scales, we can express effective energies for two cell types, denoted by *a* and *b*:

(10) $$E_{a}=K{\left(L_{cell}-L_{0,a}\right)}^{2}-\left(\epsilon_{aa}.x+\epsilon_{ab}.y\right)$$

(11) $$E_{b}=K{\left(L_{cell}-L_{0,b}\right)}^{2}-\left(\epsilon_{bb}.x+\epsilon_{ab}.y\right)$$

$\epsilon_{ab}$ denotes the adhesion energy per unit length between cells of type *a* and *b*, *x* and *y* denote the total alignment overlap with cells of the same and different types respectively, and $L_{0,a}$ and $L_{0,b}$ represent the equilibrium lengths for the different cell types.

Filopodia activity results from the interplay between active fluctuations and adhesion interactions with other filopodia. The active alignment of the heart takes place over a period of around 30 min, whereas the average binding time of filopodia is 1–5 min. In the following, we assume that the heart has enough time during the active alignment process to reach equilibrium.

To simulate the evolution and equilibration of configurations of heart cells, we use a Metropolis algorithm incorporating mechanical fluctuations induced by filopodia activity as an effective temperature; see Fig. 2 *E* and materials and methods for further details. We define cellular mismatch by identifying the fraction of cell boundaries that are not correctly aligned with their corresponding opposite cell, Fig. 2 *A*. In this definition, a perfectly aligned tissue has mismatch of 0, whereas a fully misaligned system has a mismatch of 1. The tissue mismatch is then taken as the average over all cell mismatches.

### Minimization of interfacial energy constraints is sufficient to explain cell alignment in the wild-type *Drosophila* heart

We apply our model to the developing *Drosophila* heart, where quantitative data for cell matching are available (29). As well as incorporating the effective energy differences, we need to implement within the simulation a representative pattern of cell types. In the *Drosophila* heart, Tin-positive cardioblasts express Fas3 at high levels and Svp-positive cardioblasts express Ten-m. We translate this into different adhesion energies per unit length between Tin-positive cardioblasts ($\epsilon_{TT})$, Svp-positive cardioblasts ($\epsilon_{SS})$, and between Tin- and Svp-positive cardioblasts ($\epsilon_{ST})$, similar to the outline given in Figs. 1 *B*–*E* and 2 *D*.

#### Initial configuration of the cells and initial cell alignment

By the end of dorsal closure, cardioblasts are brought into close contact. To initiate the cellular arrangement, we assume that the cells at the two ends of the heart are perfectly aligned and are at their resting length initially. To model geometric disorder, we take the length of the apical surfaces for Tin-positive and Svp-positive cardioblasts as $L_{0,T}$ and $L_{0,S}$, respectively. These lengths are simulated as Gaussian variables of mean $L_{0,T}^{mean}$ and $L_{0,S}^{mean}$ with standard deviation $L_{0,T}^{std}$ and $L_{0,S}^{std}$, extracted from experimental quantification of cell size (Fig. 2 *F*). Importantly, the experimentally measured rest lengths are used in the simulations; hence, not all cells reach the final size. Given the tissue is constrained in vivo (53), these rest lengths represent the balance between the cell intrinsic rest length and external compression (we implicitly include the effects of cell packing on individual cell size, which could in principle have different rest lengths in unconstrained environments). We construct the simulated heart as two rows of cells formed by a succession of four Tin-positive cells and two Svp-positive cells patterns, with a total number of *N*_1_ = 52 cells (number of cells in a heart row; Figs. 1 *B* and 2 *G*). Each cell length is picked as a random variable according to the rest lengths distributions (Fig. 2 *F*). However, we constrain the total lengths of the two rows to be identical (materials and methods).

#### Initial cell matching without adhesion energy

We first consider the question of whether the boundary constraints alone are sufficient to ensure robust cell matching. To answer this, we take initial conditions that mirror experimentally measured cardioblast size and position (Fig. 2 *F* and *G*) but with no adhesion energy (i.e., $\epsilon_{TT}{=\epsilon }_{SS}=\epsilon_{ST}=0$) (Fig. 2 *D*). As we take cells at their initial rest length, no equilibration is needed, and we calculate the mismatch of the initial condition. We performed 30 simulations per condition with confinement of different sizes and calculated the ensemble mismatch in cell alignment. This resulted in a mismatch increasing with system size (Fig. 2 *H*). Interestingly, we find that, at 52 cell lengths (the experimental size of the heart), we get a mismatch around 0.3, which corresponds to the mismatch value experimentally observed in the *Svp*^*−/−*^ mutants where all cells are of the same type (Fig. 2 *C*). In summary, geometric disorder, induced by cell size variability, is too large to enable precise cell matching merely by boundary constraints.

#### Calculating the mismatch with differential cell adhesion

**Figure 3:**
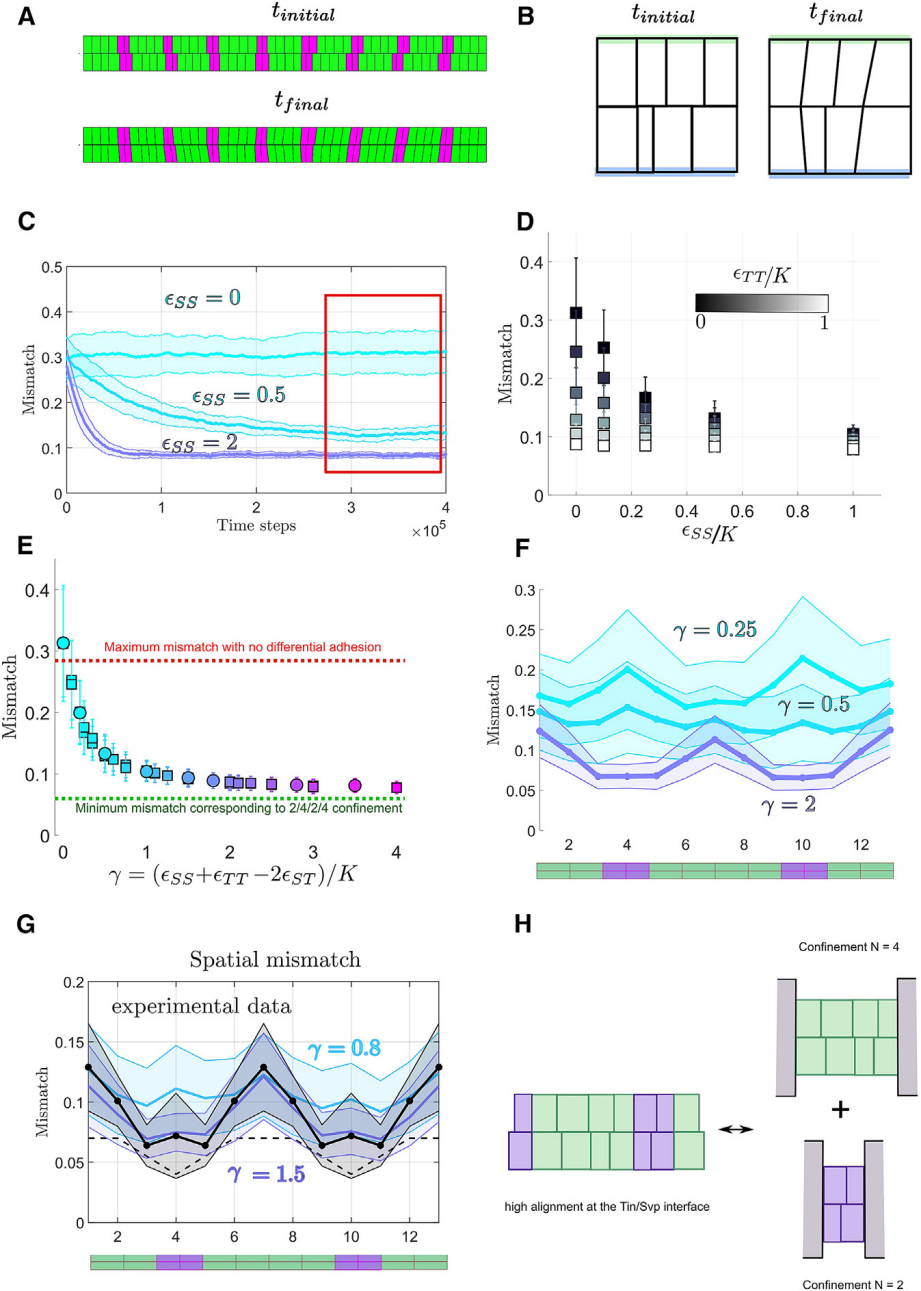
The interplay between differential adhesion, cell compressibility, and cell size variability determines cell matching. (*A*) *t*_*initial*_ corresponds to the initial configuration randomly sampled for 52 cells (four Tin/2 Svp alternated pattern) and *t*_*final*_ corresponds to the cells configuration after convergence of mismatch. (*B*) Cell lengths have changed on the cell/cell interface, whereas we still represent their initial length and position on the side not touching the interface (*blue and green interfaces*). (*C*) Ensemble averaged (N_2_ = 30 simulated embryos) mismatch evolution for different values of *ε*_SS_ and K = 1. All mismatches are calculated at steady state. (*D*) Final mismatch for different values of *ε*_SS_ and *ε*_TT_. (*E*) Final mismatch for different values of *γ*. Circles correspond to the case *ε*_ST_ = 0 and squares to *ε*_ST_ ≠0. (*F*) Spatial variation of mismatch across different cell types as depicted in the below cartoon (green = Tin-positive cardioblasts, magenta = Svp-positive cardioblasts). (*G*) Spatial variations of the mismatch in the wild type (*black*) and comparison with the simulation (*blue*, *purple*). (*H*) Sketch showing how differential adhesion aligns heterogeneous cell-cell contacts, which generates local confinement of cells.

We next investigated how the strength of selective adhesion impacts alignment. We first explored matching variations with only one cell type adhering; i.e., we varied $\epsilon_{\text{SS}}$ alone with $\epsilon_{\text{TT}}=0$ $\epsilon_{\text{ST}}=0$ (Fig. 3 *A*–*C*). We computed mismatch evolution as a function of simulation iterations and took its value once it reached a steady-state value (Fig. 3 *C*). The mismatch for each simulation was then averaged over 30 simulations to get the average final mismatch per condition. We find that the final mismatch decreases with increasing adhesion level.

We then explored two cell types with selective adhesion by using different combinations of $\epsilon_{\text{SS}}$ and $\epsilon_{\text{TT}}$ with $\epsilon_{\text{ST}}=0$ (Fig. 3 *D*). In general, increased adhesion energy led to reduced mismatch. Different combinations of $\epsilon_{\text{SS}}$ and $\epsilon_{\text{TT}}$ can lead to the same alignment. By systematically varying $\epsilon_{\text{SS}}$, $\epsilon_{\text{TT}}$, and $\epsilon_{\text{ST}}$, we see that the equilibrium mismatch is determined by the parameter

(12) $$\gamma =\frac{\left(\epsilon_{TT}-\epsilon_{ST}\right)+\left(\epsilon_{SS}-\epsilon_{ST}\right)}{K}$$

representing the competition between cells due to differential adhesion and cell compressibility (Fig. 3 *E*). Here, γ corresponds to the typical length change that adhesion differences can generate for a single cell. For γ=0μm, there is no energy cost for different cell types to have a contact interface. The mismatch corresponds to the random case simulated in Fig. 2 *H*, similar to the *Svp*^*−/−*^ mutant, where all cells are of the same type. Mismatch decreases with increasing γ, reaching a plateau around mismatch of ∼0.07 (dashed line in Fig. 3 *E*). This implies that, beyond a certain level, increasing differential adhesion only weakly improves matching. Here, γ is defined empirically, but it is consistent with the analytical mismatch found at equilibrium for a simplified 1D model consisting of two cell types (e.g., Svp and Tin), facing each with other and confined at a fixed length L, assuming the energies from Eqs. 1 and 2.

We measured spatial variations of the mismatch for different values of γ (Fig. 3 *F*). We find that the mismatch is minimal at the Tin-Svp interface, as any contact between different cell types has some adhesion energy cost for γ>0μm (Fig. 3 *G*). Alignment of cells inside a block of homogeneous cell types in the high γ limit is determined by the boundaries imposed by the highly aligned cells at the interface between different cell types. Within a region of Tin-positive cells, the level of cell mismatch converges toward the nonadhesive random case with *N* = 4 (Fig. 3 *H*). The mismatch between Svp-positive cells is similar to the random adhesion case with *N* = 2. The geometric variability in the lengths of the cell leading edges means that cells never reach zero mismatch even at very high adhesion values. Interestingly, for small values of γ, the pattern of cell mismatch is inverted, as geometric variability is higher for Svp cells.

Comparing the spatial profile of cell matching to the experimental data, we see that, for γ>0.5μm the mismatch profile agrees well with experiment, Fig. 3 *G*. Therefore, simple energy considerations, with a single fitting parameter γ, are sufficient to reproduce the wild-type matching phenotype.

### Energy scales are just sufficient to ensure robust matching

**Figure 4:**
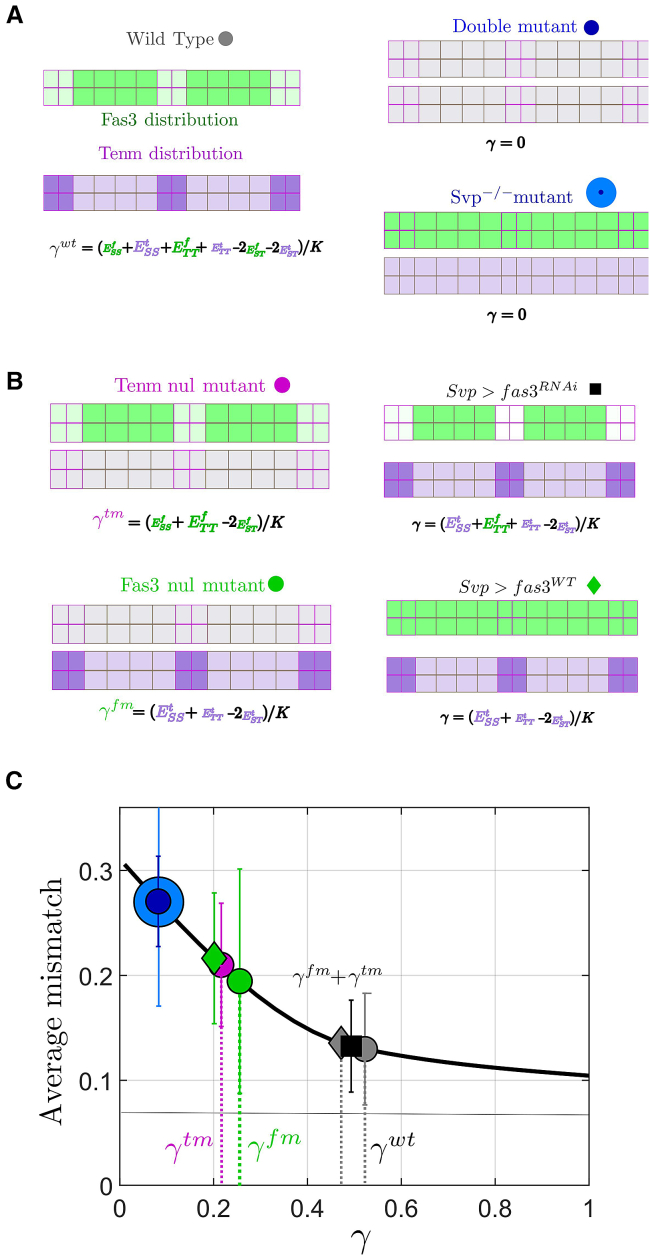
Equilibrium energy description replicates experimental observations in wild type and mutants. (*A*) Distribution of Fas3 (*green*) and Ten-m (*purple*) in wild-type embryos and the associated formula for *γ*. (*B*) Cartoons of relative interactions in different genotypes: wild type (*gray circle*); *fas3*^*−/−*^*ten-m*^*−/−*^ (*blue small circle*); *svp*^*−/−*^ (*blue circle*); *fas3*^*−/−*^ (*green circle*); *ten-m*^*−/−*^ (*purple circle*); Svp-Gal4, Fas3-UAS (*green diamond*); and Svp-Gal4 > Fas3-RNAi-UAS (*black square*). Each adhesion energy can be decomposed into two contributions from Tin and Svp-positive cells, leading to an approximation of *γ*. (*C*) Distribution of Fas3 and Ten-m mismatch in different genotypes (as defined in *B*) overlayed on the master mismatch curve of Fig. 2*E*. By placing the mismatch values on the curve, we can infer the corresponding effective *γ* on the x axis, with error bars representing the estimated error.

We do not know the specific effective energy levels associated with different adhesion interactions. To relate the values of $\epsilon_{TT},\epsilon_{SS}$, and $\epsilon_{ST}$ to the underlying dynamics, we assume that they linearly depend on the energy scales of homophilic interactions of Fas3 ($\epsilon_{aa}^{Fas3}$) and Ten-m ($\epsilon_{aa}^{Ten}$); e.g., $\epsilon_{TT}\approx \epsilon_{TT}^{Fas3}+\epsilon_{TT}^{Ten}$. In our model, if there is no differential expression pattern of Fas3 and Ten-m, then *γ* = 0 *μ*m; i.e., there is no effective adhesion energy differences between cells. For *svp*^−/−^ mutants (where all cardioblasts are Tin positive) or the double mutant *Fas3*^*−/−*^*; Ten-m*^*−/−*^ (Fig. 4 *A*), we expect γ≈0.

From previous work, the mismatch in a range of mutant conditions—such as disruption of Fas3 and Ten-m—is quantified (29). We utilized the results from Fig. 3 *E* to estimate *γ* for wild-type and mutant conditions (Fig. 4 *B*). We subsequently plotted the mismatch based on the inferred value of *γ* in a range of conditions (Fig. 4 *C*). Can our model explain the observed values for *γ* in the mutant conditions?

From Fig. 4 *C*, we have for the wild type, $\gamma_{WT}$∼0.5 μm, which is smaller than the value $\gamma_{WT}$ ∼ 1.0 μm found by fitting the experimentally measured spatial variation of mismatch (Fig. 3 *G*). However, this difference could be explained by the shallowness of the mismatch curve for *γ* > 0.5 μm. Assuming that there is no interaction between Fas3 and Ten-m, we expect that $\gamma_{WT}=\gamma_{Fas3}+\gamma_{Tenm}\text{.}$ We use the measured matching in *Fas3*^*−/−*^ and *Ten-m*^*−/−*^ mutants to estimate the contributions to $\gamma_{Tenm}$ and $\gamma_{Fas3}$, respectively. From this, we find that $\gamma_{Fas3}+\gamma_{Tenm}$ ∼$\gamma_{WT}$ ∼0.5 (Fig. 4 *C* and materials and methods).

To further test these assumptions, we considered additional mutant conditions. Overexpression of Fas3 in Svp cells should give similar value of *γ* to the *Fas3*^*−/−*^ mutant, as in both cases the differential expression pattern of Fas3 is largely lost (Fig. 4 *B*). As shown in Fig. 4 *C*, this is indeed the case. If Fas3 is reduced in Svp-positive cells, we expect $\epsilon_{SS}$ is reduced compared to wild type. However, $\epsilon_{ST}$ is also likely decreased as direct interactions between filopodia with Fas3 is reduced between different cell types. Therefore, we do not expect *γ* to differ significantly from wild type. Consistent with this, we find a *γ* similar to the wild-type case showing that $\epsilon_{ST}$ is small compared to $\epsilon_{SS}$ and $\epsilon_{TT}\text{.}$ We can map a range of observations in mutant embryos onto a single curve for the mismatch described by *γ*, suggesting our adhesion hypothesis is a reasonable approximation of the underlying dynamics driving cell matching. It is important to note that, in Fig. 4 *C*, the objective is not to determine the specific values of the six parameters $E_{SS}^{T}$, $E_{TT}^{F},E_{ST}^{F},E_{ST}^{T},E_{TT}^{T},{\text{and}\;E}_{SS}^{F}$ individually. Rather, the goal is to confirm that the *γ* values derived for each condition are consistent with this decomposition framework based on these six parameters (see materials and methods for further outline of parameter estimation).

### Robustness of cell matching to perturbations

**Figure 5:**
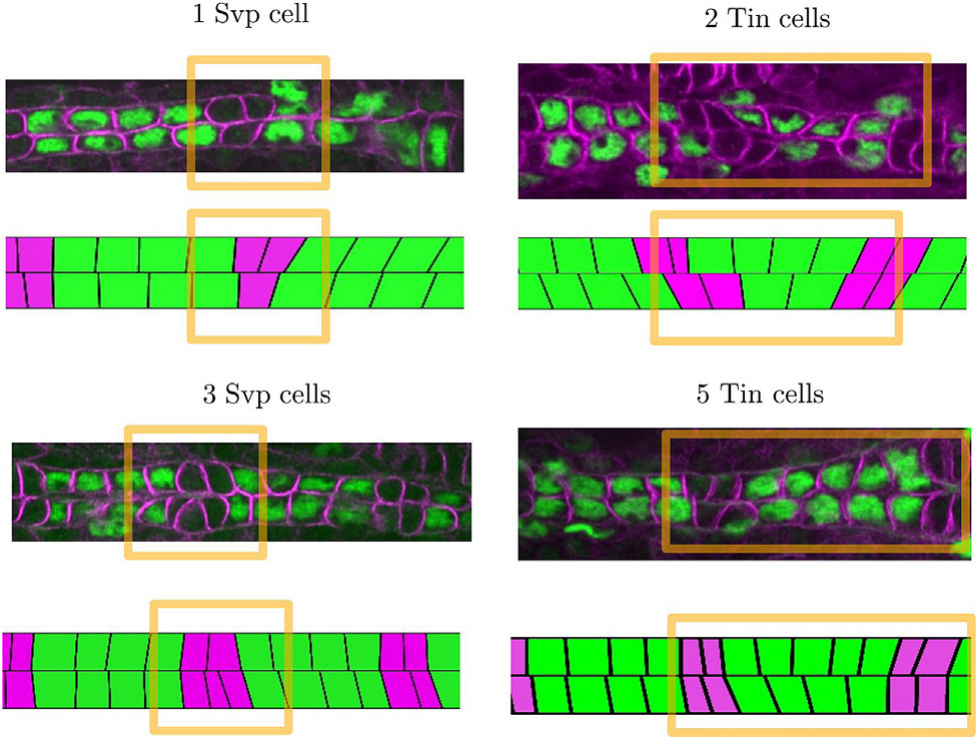
Matching correction of local defects. Various types of experimentally observed cell number defects in wild-type embryonic hearts. Differential adhesion can partially compensate these defects by deforming cells in the range of *γ* ≈ 0.5 found in Fig. 4.

Here, we probe the robustness of the system to perturbations in cell number by considering variations in cell type specification. Within wild-type embryo populations, we observe variability in the spatial pattern of cardioblast specification. For example, we have observed embryos with three and five Tin-positive cardioblasts in a sector, as well as a solitary Svp-positive cardioblast, and an additional one (Fig. 5). Even when cell number is perturbed, we observe that the boundaries between different cell types are typically well defined. To test the model, we implemented different initial patterns of cardioblasts but otherwise kept parameters identical to the wild-type scenario with *γ* = 0.5. The system equilibrates to a state with clear boundaries between the different cell types and cells deform to align boundaries. This demonstrates that having two complementary adhesion processes is robust to perturbations in the structure of the heart as it develops.

### Dynamic vertex model of cell matching

**Figure 6:**
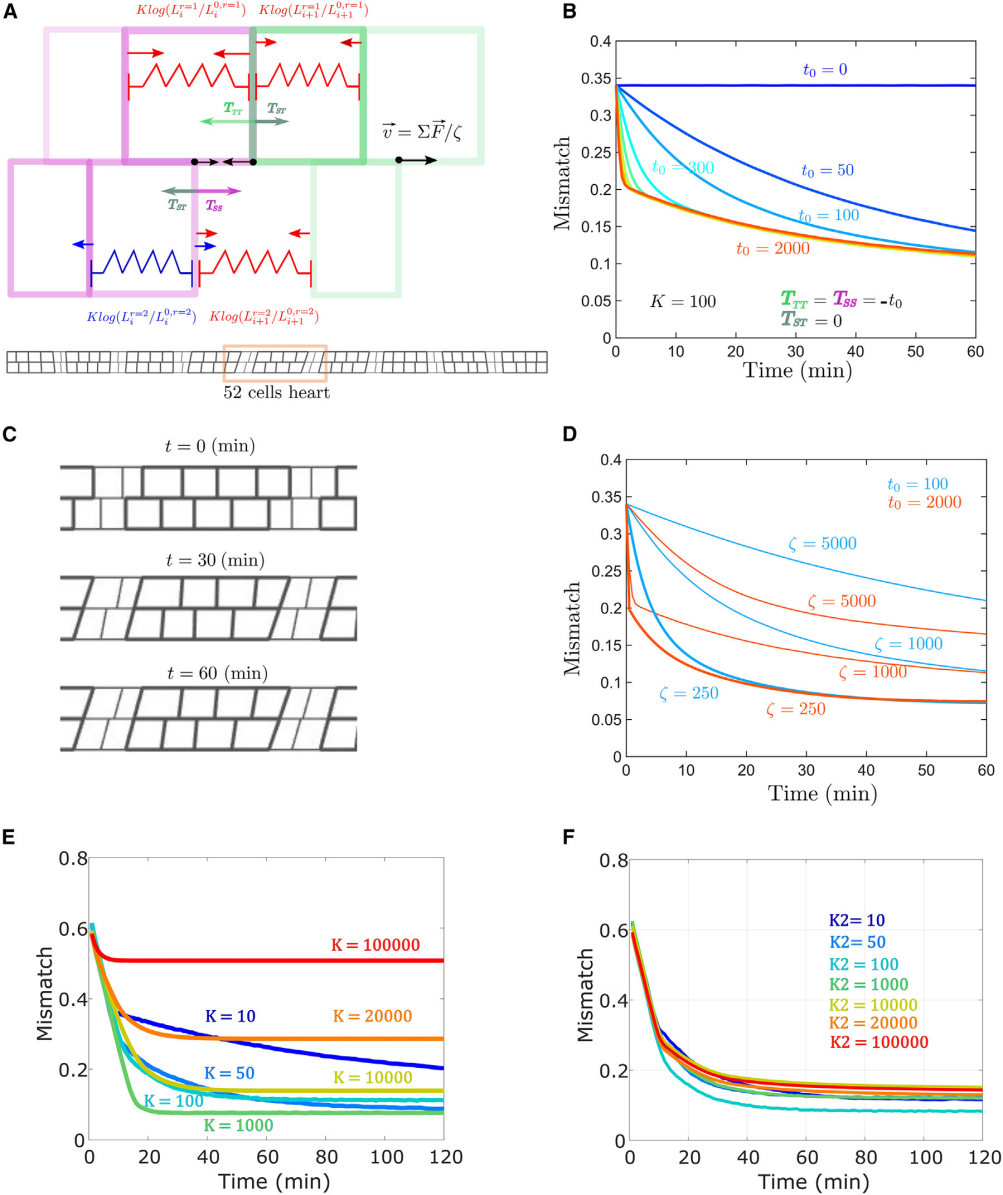
Simulating cell-matching dynamics. (*A*) Vertex-model implementation of interface dynamics. Each cell-cell lateral boundary on a row is defined by a vertex point. Elastic forces are represented in red/blue depending on whether cells are stretched/compressed. Each cell-cell interface section between the two facing rows has a negative tension, which acts on the vertices applying forces represented in magenta/green/gray depending on the interface type. The sum of these contributions divided by a friction coefficient, *ξ*, gives the vertex velocity. (*B*) We evolve the system (Materials and Methods) and compute the ensemble averaged mismatch over time (*N* = 30). We vary the tension ${\;t}_{0}$ between 0 and 2000 while K is fixed to 100 and *ξ* = 1000. (*C*) Spatial alignment for 0, 30, and 60 min for ${\;t}_{0\;}=2000\text{,}$ K = 100, and *ξ* = 1000. (*D*) Adhesion-independent cell alignment between cells of the same type depends on the friction coefficient *ξ*. Results shown for two different tensions, t_0_. (*E*) The time taken to reach minimal mismatch within the vertex model with different *K* applied the same to all cells within each simulation. (*F*) As in (*E*), except one cell kept at K = 100 and the other cell’s stiffness varied over orders of magnitude.

To evaluate how differential adhesion modulates cell-matching dynamics, we used a vertex approach to model the time evolution of vertices at the interface between the two facing rows of cells (Fig. 6 *A*; see materials and methods). By analogy with the equilibrium energy model used previously, vertices are submitted to elastic forces corresponding to effective elasticity and adhesion forces in the cell. We assume that adhesion produces a negative tension, which extends cell-cell contact interfaces. The sum of these contributions results in a force that, when divided by an effective friction coefficient, gives the vertex velocity. We explore the simple case of equal tensions, ${\;T}_{\text{SS}}{=T}_{\text{TT}}{=-t}_{0}$ and ${\;T}_{\text{ST}}=0$ with a fixed K and friction coefficient, *ξ*. Varying $t_{0}$, keeping other parameters fixed, shows a lower limit to cell-matching accuracy, as the time evolution of cell matching collapses onto a single curve for large $t_{0}$ (Fig. 6 *B*). This limit is consistent with our equilibrium model. At high adhesion differences, the cell mismatch first undergoes a rapid decrease, which is adhesion dependent. This corresponds to the phase of heterogeneous cell-type boundaries shrinking. Below a mismatch of ∼0.2, the boundaries between cell types are perfectly aligned (Fig. 6 *C*). The mismatch between cells of the same type decreases with slower, adhesion-independent dynamics that are friction dependent (Fig. 6 *D*). Overall, we see that dynamically incorporating differential adhesion gives similar results to our equilibrium approach, supporting our above assumptions.

### Role of stiffness in cell matching

Finally, we explored in more detail the role of cell rigidity in the ability of cells to reliably match. We varied the global cell rigidity across five orders of magnitude and analyzed the reduction in cell mismatch with time within our vertex model (Fig. 6 *E*). At high K, the mismatch saturates at a value near its initial state, as the maximal segment deformations resulting from the equilibrium between elastic and adhesive forces cannot fully compensate for misalignment. For intermediate K, the mismatch reaches a minimal value within the simulation time frame. At low K, mismatch relaxation takes too long relative to the typical morphogenetic time window allowed for the matching process (∼1 h). Therefore, within experimentally relevant windows, there is an intermediate range of K optimal for ensuring cell alignment. To further probe this relationship, we maintained one cell type at intermediate K but altered the rigidity in the other cell type. We observed that the system was still able to reach a minimal state (Fig. 6 *F*).

## Discussion

Our model, although grounded in simple biophysical principles, effectively accounts for a wide range of mutant phenotypes. The model enables exploration of how periodic differential adhesion patterns of cells can counterbalance initial geometrical disorder in cells. Future studies could investigate the dynamics of alignment, examining factors like dissipation, including cortical viscosity and tissue friction, to see how closely experimental data can match the model’s detailed predictions. In particular, how does extension into 2D and 3D impact the model behavior, including the importance of cell volume conservation?

Even though biological systems are inherently out of equilibrium, we have demonstrated that an equilibrium energy argument is consistent with experimental observation of cell matching in the heart. This is consistent with the rapid dynamics of filopodia compared to the time of heart cells’ final alignment. The filopodia dynamics relate to the system’s effective temperature. The idea of effective temperatures in developing systems has also recently been used to describe jamming transitions during zebrafish development (54,55). Our model shows that cell matching results from the interplay between cell-cell interactions (mediated by filopodia and specific adhesion molecules) and cell deformability. According to our model, the level of differential adhesion found in the wild-type heart is close to the minimal level required to obtain a well-aligned heart as the value *γ* = 0.5*μ*m corresponds to the point where the mismatch curve in function of *γ* starts to flatten. We find that Fas3 and Ten-m contribute almost equally to the matching process. Furthermore, we demonstrate than increasing differential adhesion indefinitely does not accelerate the matching process beyond a certain point as the final alignment phase is adhesion independent.

A key result of our model is that short-range constraints (i.e., adhesion differences between two cell types) can propagate long-range order. We show that generating periodic segments of different adhesion molecules 1) aligns these segments at their interfaces and 2) alignment inside each segment is due to geometric confinement and decreases with the segment size. Most previous theoretical work on the role of differential adhesion in tissue shaping has focused on how two (or more) distinct tissues can define clear boundaries (25). Our results are complementary to such work and demonstrate that differential adhesion is a viable mechanism for aligning cells within a single tissue. Recent work has also highlighted how morphogen patterning of tissues coupled with mechanical feedback can build long-range tissue architecture (56). It will be interesting to explore how spatial information and mechanical feedback interplay in different tissues to ensure tight domain formation.

Here, we have not considered long-range actin structures that span multiple cells. These have been observed during heart closure and appear to play an important role in cardioblast migration (48). We have not considered these structures here explicitly as they are not at the leading edges of the cells where cell matching first occurs. Such structures could play an important role in integrating mechanical information during closure. Work in other systems has highlighted that local variations in the cellular microenvironment need to be buffered at tissue scales (57) and that such noise sets a limit of the precision of organ development. One possible role for the long-range actin structures here may be in buffering cell-to-cell mechanical variations as they average forces over multiple cell diameters. Future work could ablate these structures as the heart closes and assay changes in cell matching.

Our work has potential application to other biological systems. During neurogenesis—where precise connections between neurons are required—Notch signaling (involved in lateral inhibition) has recently been shown to regulate neuronal wiring (13). Formation of the vasculature requires cell migration to precise locations and this is mediated by filopodia protrusions and lateral inhibition (58). During wound healing, cells need to repair wounds by forming precise connections. However, how processes at a single-cell level are integrated to ensure long-range precise cell matching remains an open question (59). In our model, we do not consider heterogeneities in the mechanical properties. In other systems, such as the brain, tuning of tissue stiffness ensures precise cell matching (60). More generally, coordinated cell migration can be directed by tissue stiffening (61). It would be possible to test the role of mechanical feedback in our model by adding viscoelastic relaxation of the cell lengths within a vertex-model framework as described in Fig. 6. Our results on the rigidity dependence (Fig. 6 *E* and *F*) suggest that changes to cell stiffness can impact matching. Through targeted drug or RNAi perturbation, it will be interesting to explore whether this can be tested in vivo.

## Acknowledgments

This work was supported by the 10.13039/501100000741University of Warwick, 10.13039/501100003043EMBO 10.13039/100008086Global Investigator, Singapore Ministry of Education Academic Research Fund Tier 2 grant (MOE2018-T2-2-135), 10.13039/501100001381Singapore National Research Foundation Fellowship (NRF2012NRF-NRFF001-094), 10.13039/100004412HFSP Young Investigator grant (RGY0083/2016), and 10.13039/501100000274British Heart Foundation research grant (PG/22/11031), all awarded to T.E.S. Tinman antibody was a gift from Manfred Frasch.

## Author contributions

S.T. and T.E.S. designed the project with input from S.Z. S.T. and M.S. performed the simulations and modeling. S.Z. provided all experimental data. S.T. and T.E.S. wrote the manuscript, with contributions from M.S. and S.Z.

## Declaration of interests

The authors declare no competing interests.

## Supporting Material

Document S1. Figure S1

Document S2. Article plus supporting material

## References

[1] Lecuit T., Le Goff L. (2007). Orchestrating size and shape during morphogenesis. Nature.

[2] Heisenberg C.P., Bellaïche Y. (2013). Forces in tissue morphogenesis and patterning. Cell.

[3] Collinet C., Rauzi M., Lecuit T. (2015). Local and tissue-scale forces drive oriented junction growth during tissue extension. Nat. Cell Biol..

[4] Pinheiro D., Bellaïche Y. (2018). Mechanical Force-Driven Adherens Junction Remodeling and Epithelial Dynamics. Dev. Cell.

[5] Athilingam T., Tiwari P., Saunders T.E. (2021). Mechanics of epidermal morphogenesis in the Drosophila pupa. Semin. Cell Dev. Biol..

[6] Olivotto I., Cecchi F., Yacoub M.H. (2009). Developmental origins of hypertrophic cardiomyopathy phenotypes: a unifying hypothesis. Nat. Rev. Cardiol..

[7] Bruneau B.G. (2020). The developing heart: from The Wizard of Oz to congenital heart disease. Development.

[8] Som P.M., Naidich T.P. (2014). Illustrated review of the embryology and development of the facial region, part 2: Late development of the fetal face and changes in the face from the newborn to adulthood. AJNR. Am. J. Neuroradiol..

[9] Zhang S., Saunders T. (2021). Mechanical processes underlying precise and robust cell matching. Semin. Cell Dev. Biol..

[10] Dixon M.J., Marazita M.L., Murray J.C. (2011). Cleft lip and palate: understanding genetic and environmental influences. Nat. Rev. Genet..

[11] Clandinin T.R., Zipursky S.L. (2002). Making connections in the fly visual system. Neuron.

[12] Sanes J.R., Yamagata M. (2009). Many paths to synaptic specificity. Annu. Rev. Cell Dev. Biol..

[13] Pinto-Teixeira F., Koo C., Desplan C. (2018). Development of Concurrent Retinotopic Maps in the Fly Motion Detection Circuit. Cell.

[14] Takeichi M. (2007). The cadherin superfamily in neuronal connections and interactions. Nat. Rev. Neurosci..

[15] Hong W., Mosca T.J., Luo L. (2012). Teneurins instruct synaptic partner matching in an olfactory map. Nature.

[16] Maness P.F., Schachner M. (2007). Neural recognition molecules of the immunoglobulin superfamily: signaling transducers of axon guidance and neuronal migration. Nat. Neurosci..

[17] Berns D.S., DeNardo L.A., Luo L. (2018). Teneurin-3 controls topographic circuit assembly in the hippocampus. Nature.

[18] Harada T., Harada C., Parada L.F. (2007). Molecular regulation of visual system development: more than meets the eye. Genes Dev..

[19] Kumar J.P. (2012). Building an ommatidium one cell at a time. Dev. Dyn..

[20] Wartlick O., Jülicher F., Gonzalez-Gaitan M. (2014). Growth control by a moving morphogen gradient during Drosophila eye development. Development.

[21] Olson E.N. (2006). Gene regulatory networks in the evolution and development of the heart. Science.

[22] Hunter G.L., Hadjivasiliou Z., Baum B. (2016). Coordinated control of Notch/Delta signalling and cell cycle progression drives lateral inhibition-mediated tissue patterning. Development.

[23] Shaya O., Binshtok U., Sprinzak D. (2017). Cell-Cell Contact Area Affects Notch Signaling and Notch-Dependent Patterning. Dev. Cell.

[24] Schotz E.M., Burdine R.D., Foty R.A. (2008). Quantitative differences in tissue surface tension influence zebrafish germ layer positioning. HFSP J..

[25] Manning M.L., Foty R.A., Schoetz E.M. (2010). Coaction of intercellular adhesion and cortical tension specifies tissue surface tension. Proc. Natl. Acad. Sci. USA.

[26] Foty R.A., Pfleger C.M., Steinberg M.S. (1996). Surface tensions of embryonic tissues predict their mutual envelopment behavior. Development.

[27] Foty R.A., Steinberg M.S. (2005). The differential adhesion hypothesis: a direct evaluation. Dev. Biol..

[28] Iruela-Arispe M.L., Beitel G.J. (2013). Tubulogenesis. Development.

[29] Zhang S., Amourda C., Saunders T.E. (2018). Selective Filopodia Adhesion Ensures Robust Cell Matching in the Drosophila Heart. Dev. Cell.

[30] Compernolle V., Brusselmans K., Carmeliet P. (2003). Cardia bifida, defective heart development and abnormal neural crest migration in embryos lacking hypoxia-inducible factor-1alpha. Cardiovasc. Res..

[31] Gibson M.C., Patel A.B., Perrimon N. (2006). The emergence of geometric order in proliferating metazoan epithelia. Nature.

[32] Umetsu D., Aigouy B., Dahmann C. (2014). Local increases in mechanical tension shape compartment boundaries by biasing cell intercalations. Curr. Biol..

[33] Hamant O., Heisler M.G., Traas J. (2008). Developmental patterning by mechanical signals in Arabidopsis. Science.

[34] Taniguchi K., Maeda R., Matsuno K. (2011). Chirality in planar cell shape contributes to left-right asymmetric epithelial morphogenesis. Science.

[35] Hervieux N., Dumond M., Olivier H. (2016). A Mechanical Feedback Restricts Sepal Growth and Shape in Arabidopsis. Curr. Biol..

[36] Le Garrec J.F., Dominguez J.N., Meilhac S.M. (2017). A predictive model of asymmetric morphogenesis from 3D reconstructions of mouse heart looping dynamics. Elife.

[37] Hamant O., Saunders T.E. (2020). Shaping Organs: Shared Structural Principles Across Kingdoms. Annu. Rev. Cell Dev. Biol..

[38] Matejcic M., Salbreux G., Norden C. (2018). A non-cell-autonomous actin redistribution enables isotropic retinal growth. PLoS Biol..

[39] Collier J.R., Monk N.A., Lewis J.H. (1996). Pattern formation by lateral inhibition with feedback: a mathematical model of delta-notch intercellular signalling. J. Theor. Biol..

[40] Bi D., Lopez J.H., Manning M.L. (2015). A density-independent rigidity transition in biological tissues. Nat. Phys..

[41] Kim S., Cassidy J.J., Hilgenfeldt S. (2016). Hexagonal Patterning of the Insect Compound Eye: Facet Area Variation, Defects, and Disorder. Biophys. J..

[42] Lou Y., Rupprecht J.F., Saunders T.E. (2023). Curvature-Induced Cell Rearrangements in Biological Tissues. Phys. Rev. Lett..

[43] Hayashi T., Carthew R.W. (2004). Surface mechanics mediate pattern formation in the developing retina. Nature.

[44] Zhang S., Teng X., Saunders T.E. (2020). Periodic Oscillations of Myosin-II Mechanically Proofread Cell-Cell Connections to Ensure Robust Formation of the Cardiac Vessel. Curr. Biol..

[45] Zhang S., Saunders T.E. (2021). Protocol for batch imaging and quantification of cellular mismatch during Drosophila embryonic heart formation. STAR Protoc..

[46] Bodmer R., Frasch M. (2010). Heart Development and Regeneration.

[47] Haack T., Schneider M., Renault A.D. (2014). Drosophila heart cell movement to the midline occurs through both cell autonomous migration and dorsal closure. Dev. Biol..

[48] Balaghi N., Erdemci-Tandogan G., Fernandez-Gonzalez R. (2023). Myosin waves and a mechanical asymmetry guide the oscillatory migration of Drosophila cardiac progenitors. Dev. Cell.

[49] Krauth W. (2006).

[50] Metropolis N., Rosenbluth A.W., Teller E. (1953). Equation of State Calculations by Fast Computing Machines. J. Chem. Phys..

[51] Kuntz S.G., Eisen M.B. (2014). Drosophila embryogenesis scales uniformly across temperature in developmentally diverse species. PLoS Genet..

[52] Chong J., Amourda C., Saunders T.E. (2018). Temporal development of Drosophila embryos is highly robust across a wide temperature range. J. R. Soc. Interface.

[53] Tiwari P., Rengarajan H., Saunders T.E. (2021). Scaling of internal organs during Drosophila embryonic development. Biophys. J..

[54] Atia L., Bi D., Fredberg J.J. (2018). Geometric constraints during epithelial jamming. Nat. Phys..

[55] Atia L., Fredberg J.J., Pegoraro A.F. (2021). Are cell jamming and unjamming essential in tissue development?. Cells Dev..

[56] Yang S., Palmquist K.H., Rodrigues A.R. (2023). Morphogens enable interacting supracellular phases that generate organ architecture. Science.

[57] Srivastava V., Hu J.L., Gartner Z.J. (2023). Configurational entropy is an intrinsic driver of tissue structural heterogeneity. bioRxiv.

[58] Bentley K., Mariggi G., Bates P.A. (2009). Tipping the balance: robustness of tip cell selection, migration and fusion in angiogenesis. PLoS Comput. Biol..

[59] Sonnemann K.J., Bement W.M. (2011). Wound repair: toward understanding and integration of single-cell and multicellular wound responses. Annu. Rev. Cell Dev. Biol..

[60] Koser D.E., Thompson A.J., Franze K. (2016). Mechanosensing is critical for axon growth in the developing brain. Nat. Neurosci..

[61] Barriga E.H., Franze K., Mayor R. (2018). Tissue stiffening coordinates morphogenesis by triggering collective cell migration in vivo. Nature.

