# Supplementary material for: Interfacial energy constraints are sufficient to align cells over large distances
Source: Biophys J. 2025 Mar 12;124(6):1011–23. doi: 10.1016/j.bpj.2025.02.011 (PMC11947472; doi:10.1016/j.bpj.2025.02.011)
Supplement: Document S1. Figure S1 [file mmc1.pdf]

Biophysical Journal, Volume *124*

Supplemental information

Interfacial energy constraints are sufficient to align cells over large distances

Sham Tlili, Murat Shagirov, Shaobo Zhang, and Timothy E. Saunders

Interfacial energy constraints are sufficient to align cells over large distances

Supplementary Information

S. Tlili[1,2], M. Shagirov[1], S. Zhang[1] and T. E. Saunders[1,3,4,5,#]

[1] Mechanobiology Institute, National University of Singapore, Singapore

[2] Aix-Marseille University, CNRS, UMR 7288, IBDM, Turing Center for Living Systems, Marseille, France

[3] Department of Biological Sciences, National University of Singapore, Singapore

[4] Institute of Molecular and Cell Biology, A*Star, Singapore

[5] Warwick Medical School, University of Warwick, United Kingdom

[#] Correspondence: timothy.saunders@warwick.ac.uk

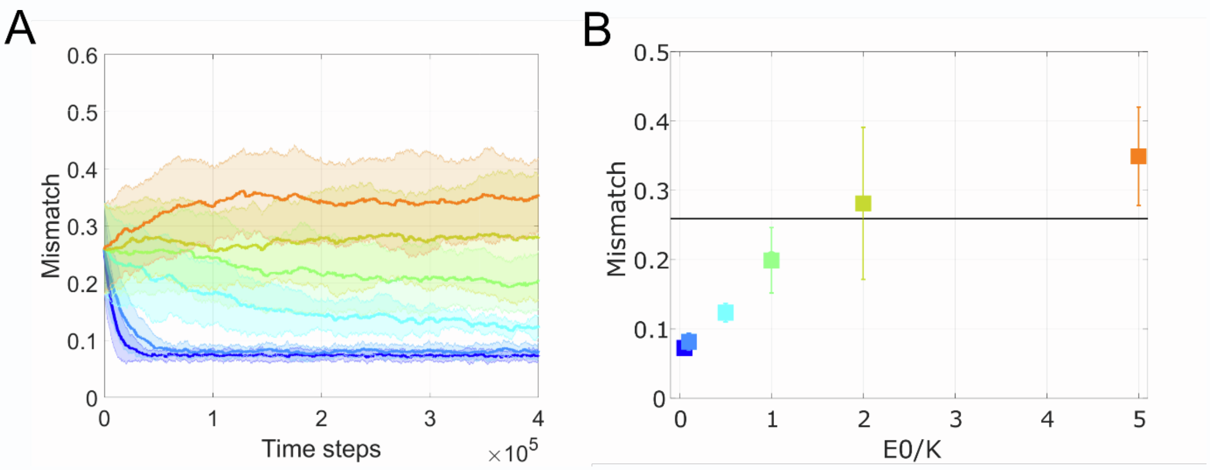

Fig. Supp 1: Impact of the effective temperature E_0 on the final mismatch obtained in Monte-Carlo simulations. $\frac{E_0}{K}$ is varied between 0.05 and 5 (0.05 in dark blue, 0.1 in blue, 0.5 in light blue, 1 in green, 2 in yellow and 5 in red) taking $\gamma = 1\mu m$ (averaged over 10 simulations). (A) Evolution of the mismatch with simulations iterations. (B) Final mismatch obtained depending on $\frac{E_0}{K}$. The average initial mismatch is represented by the black line. Choosing a low enough E_0 enables to recover the low temperature limit for the mismatch while increasing too much E_0 adds additional disorder in the system and increases the final mismatch compared to the initial state.
